# Atherosclerosis features in rheumatic diseases – focus on peripheral artery disease

**DOI:** 10.3389/fimmu.2026.1814953

**Published:** 2026-04-22

**Authors:** Alexandr Ceasovschih, Alina Dima, Javier Rodriguez-Carrio, Anastasia Balta, Raluca-Elena Alexa, Bianca Codrina Morarasu, Catalina Lionte, Fotios Barkas, Maciej Banach, Victorita Sorodoc, Laurentiu Sorodoc

**Affiliations:** 1 Grigore T. Popa University of Medicine and Pharmacy, Iasi, Romania; 2 Clinical Department 5, Faculty of Medicine, Carol Davila University of Medicine and Pharmacy, Bucharest, Romania; 3 Department of Rheumatology, Colentina Clinical Hospital, Bucharest, Romania; 4 Area of Immunology, Department of Functional Biology, Faculty of Medicine, University of Oviedo, Oviedo, Spain; 5 Basic and Translational Research on Inflammatory Diseases, Department of Metabolism, Instituto de Investigación Sanitaria del Principado de Asturias (ISPA), Oviedo, Spain; 6 Department of Internal Medicine, Faculty of Medicine, School of Health Sciences, University of Ioannina, Ioannina, Greece; 7 Faculty of Medicine, John Paul II Catholic University of Lublin, Lublin, Poland; 8 Department of Preventive Cardiology and Lipidology, Medical University of Lodz (MUL), Lodz, Poland; 9 Ciccarone Center for the Prevention of Cardiovascular Disease, Johns Hopkins University School of Medicine, Baltimore MD, United States

**Keywords:** atherosclerosis, autoimmune diseases, peripheral artery disease, rheumatic and musculoskeletal diseases, rheumatoid arthritis, systemic lupus erythematosus

## Abstract

Rheumatic and musculoskeletal diseases (RMDs) confer an increased cardiovascular risk beyond traditional factors, with peripheral artery disease (PAD) being an important source of morbidity and disability in these patients. This review summarizes current evidence on PAD across RMDs, including rheumatoid arthritis, systemic lupus erythematosus, antiphospholipid syndrome, systemic sclerosis, polymyalgia rheumatica, psoriatic arthritis, and primary Sjögren’s syndrome. Physiopathological mechanisms involved include persistent inflammation, immune dysregulation, and the presence of pathogenic autoantibodies. Protective humoral responses have also been linked to reduced CV risk and may serve as future biomarkers. Clinical studies reveal variable PAD prevalence across diseases but consistent high underdiagnosis. Optimal management requires aggressive CV risk control, including lipid-lowering, immunomodulatory, and biologic therapies. This review underscores PAD as a distinct and clinically relevant manifestation of systemic autoimmunity, calling for targeted screening and prevention strategies in rheumatic populations.

## 1 Introduction

Atherosclerosis is a chronic, progressive, inflammatory disease of the arterial wall that underlies major cardiovascular diseases (CVD), including coronary artery disease, ischemic stroke, and peripheral artery disease (PAD) (1, 2). As the leading cause of death globally, CVD impose a substantial burden, with PAD alone affecting over 120 million individuals and contributing significantly to functional impairment and limb ischemia (3).

Rheumatic and musculoskeletal diseases (RMDs) have been associated with a high burden of atherosclerosis. Nevertheless, the majority of studies and current recommendations have solely focused on coronary and cerebrovascular outcomes, while PAD-specific evidence remains limited and heterogeneous (4). PAD, although it presents with a high morbidity and is prognostically important, has received little specific attention – for instance, it is not addressed in the 2022 EULAR recommendations for CVD risk management (5).

This review focuses on PAD in autoimmune RMDs, integrating mechanistic insights and evidence from clinical studies, which are summarized in dedicated tables, and discussing their clinical and therapeutic implications.

## 2 Inflammation as a driver of atherosclerosis: mechanisms and clinical relevance

**Figure 1:**
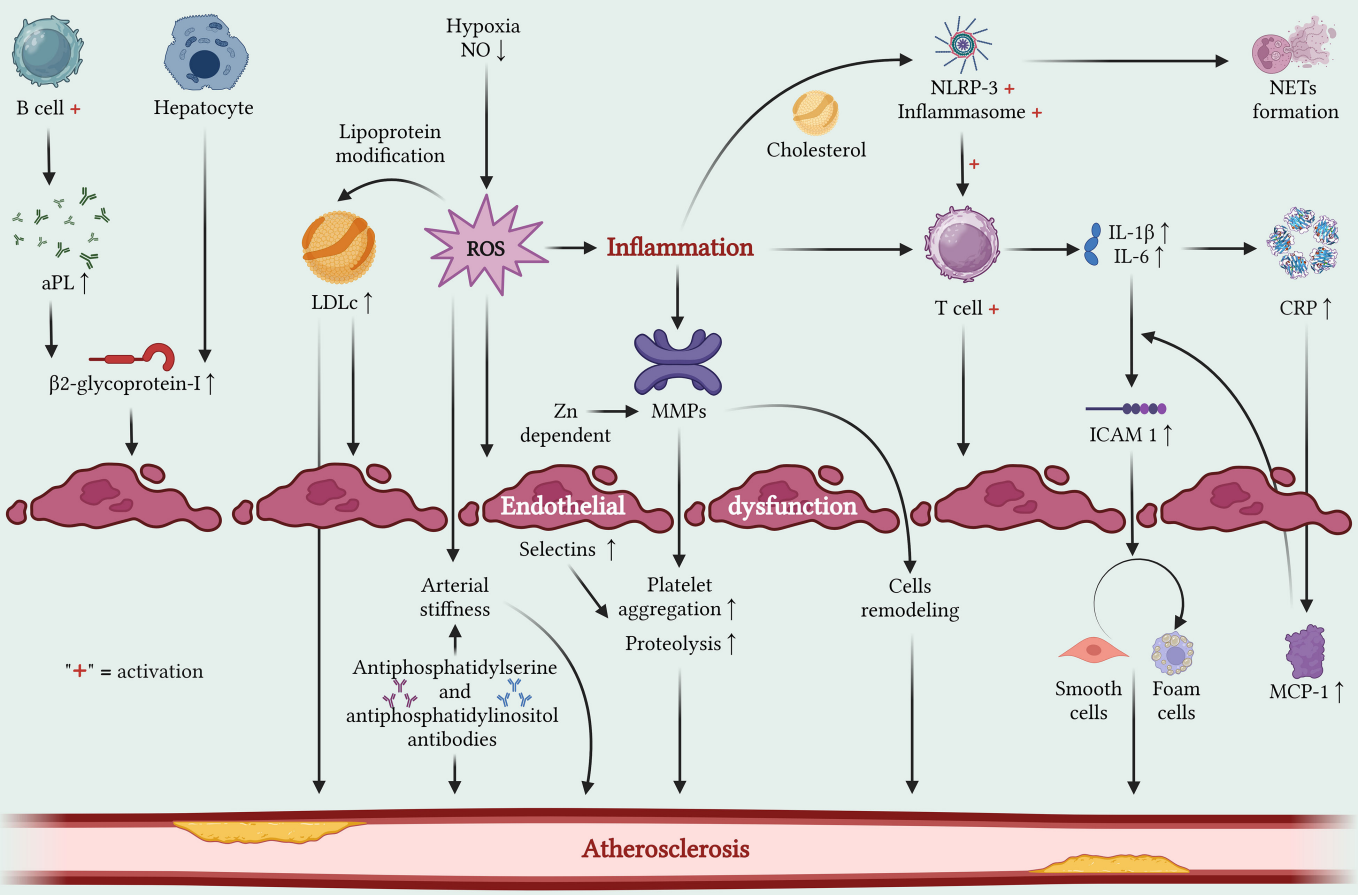
Inflammatory circuits and atherosclerosis progression in RMDs. A number of inflammatory pathways have been linked to atherosclerosis progression, also leading to arterial stiffness and/or endothelial dysfunction. These mechanisms ranged from pro-inflammatory production, lipoprotein modification, humoral responses, and T-cell activation. Molecular products of these events have been demonstrated to ultimately guide endothelial damage, and maladaptive remodeling, thus triggering atherosclerosis progression. Translational and clinical evidence have demonstrated that therapeutic manipulation of these circuits dampens atherosclerosis progression and thus, CV risk. Created in BioRender. Ceasovschih, A. (2026) https://BioRender.com/3ap6o9m.

Inflammation plays a central role in atherosclerosis (**Figure 1**), the main pathogenic mechanism underlying PAD. Acting as both trigger and aggravating factor, inflammation initiates, expands, and sustains endothelial injury, oxidative stress, and lipid accumulation within the arterial wall (6). Endothelial dysfunction, characterized by reduced nitric oxide bioavailability and impaired vascular repair, is an early hallmark of atherogenesis (7, 8). This process is sustained by activation of the innate and adaptive immune systems. Macrophages and T lymphocytes infiltrate the vascular wall and release proinflammatory cytokines, particularly interleukin (IL)-1β and IL-6, which amplify local and systemic inflammation (9, 10). Cholesterol crystal deposition further enhances this response through activation of the NOD-like receptor family pyrin domain containing 3 (NLRP3) inflammasome, leading to increased IL-1β and IL-18 production and downstream neutrophil extracellular trap formation, which perpetuates macrophage activation (11). In parallel, matrix metalloproteinases (MMPs) contribute to extracellular matrix degradation, vascular remodeling, and plaque instability (12, 13).

From a clinical perspective, inflammatory pathways have been linked to cardiovascular (CV) risk beyond traditional factors. In a large prospective cohort by Ridker et al., elevated high-sensitivity C-reactive protein (hsCRP) levels identified individuals with residual inflammatory risk, independent of lipid levels (14). However, IL-6 appears more consistently associated with atherosclerotic burden and progression. Population-based imaging studies, including the Tromsø Study and the Cardiovascular Health Study, have demonstrated independent associations between IL-6 levels and plaque progression, severity, and vulnerability, even after adjustment for traditional CV risk factors (15, 16). In addition, IL-6 has been shown to improve risk stratification beyond hsCRP, identifying individuals at higher CV risk despite similar hsCRP levels, and has been more strongly associated with CV event rates (17). Importantly, evidence specific to PAD, although still limited, points in the same direction. In a secondary analysis of the Cardiovascular Inflammation Reduction Trial (CIRT), elevated baseline IL-6 levels were independently associated with incident PAD, with subjects above the median demonstrating more than a twofold higher risk (18).

In RMDs, where systemic inflammation is chronic, hsCRP levels are frequently elevated, reducing their specificity for vascular risk assessment. Persistent inflammatory activity may nevertheless contribute to increased CV risk in these populations (19, 20). However, the clinical use of IL-6 remains limited by biological variability, short half-life, and lack of standardized assays, which complicate its routine measurement (17). Moreover, PAD-specific evidence remains scarce, and the direct applicability of these biomarkers to PAD requires further investigation.

## 3 Autoimmunity and atherosclerosis in RMDs

### 3.1 General considerations

RMDs are associated with increased CV morbidity that is not fully explained by traditional risk factors. This excess risk is largely attributed to chronic systemic inflammation and immune-mediated vascular injury, which promote endothelial dysfunction and accelerate atherosclerosis (21). In addition to conventional risk factors, non-traditional mechanisms such as persistent inflammatory signaling, immune complex formation, complement activation, and endothelial dysfunction further contribute to vascular damage in these conditions (21).

This discrepancy between lipid levels and CV risk is exemplified by a phenomenon called the “lipid paradox”, observed in RMDs, particularly in rheumatoid arthritis (RA) (22). Studies have demonstrated that inflammation is associated with decreased levels of total cholesterol, low-density lipoprotein (LDL) cholesterol, and high-density lipoprotein (HDL) cholesterol despite increased CV risk, an effect likely attributed to inflammation-driven alterations in lipoprotein composition and metabolism (23, 24). This phenomenon may complicate CV risk assessment, as patients with apparently low lipid levels may still be at increased risk and could be under-recognized if lipid values are interpreted in isolation (22, 24).

### 3.2 Autoimmunity against modified lipoproteins

Beyond the disease-specific autoantibodies found in RMDs, research has identified antibodies directly implicated in atherosclerosis. Current evidence supports the concept that atherosclerosis is a chronic immune-mediated disease involving lymphocytes and macrophages (25). Oxidized low-density lipoprotein (oxLDL), generated through enzymatic modification, increases the expression of adhesion molecules on endothelial cells and promotes recruitment of immune cells, contributing to plaque development (26). Moreover, oxLDL is taken up by macrophages, promoting their conversion into foam cells, a key element in the atherosclerotic process (25).

As foam cells accumulate, inflammatory pathways further contribute to disease progression. IL-1 activation drives IL-6–mediated amplification and an acute-phase response, integrating immune activation with traditional CV risk factors (27, 28).

Autoantibodies against oxLDL have been detected in patients with atherosclerosis and autoimmune diseases such as systemic lupus erythematosus (SLE), systemic sclerosis (SSc), and systemic vasculitides (25). Their clinical associations, however, appear heterogeneous. In a cohort including SLE, RA, and SSc patients, SSc patients with high anti-oxLDL titers displayed higher immunoglobulin concentrations and increased disease activity, yet a lower prevalence of carotid and/or femoral plaques. These results were not uniform across the remaining conditions (29). Similarly, in psoriasis, oxLDL antibody titers correlated positively with disease severity and inflammatory markers (30).

Therapeutically, the monoclonal antibody MLDL1278a, or Orticumab, which targets oxLDL (MDA-modified human ApoB100), exerts an anti-inflammatory effect by regulating Syk and p38 MAPK phosphorylation, and by modulating nuclear factor kappa B (NF-κB) activity (26). NF-κB regulates immunity, inflammation, and cell survival, and its activation by TNF and IL-1 links inflammatory and rheumatologic pathways with CVD (31).

Interestingly, autoantibodies against ApoB epitopes of oxLDL are abundant in atherosclerotic plaques (32), and higher circulating levels have been associated with lower CV risk (33).

### 3.3 Autoimmunity against native lipoproteins

With traditional CV risk factors unable to fully explain the excess morbidity and mortality in autoimmune populations, several studies have reported the presence of autoantibodies against native lipoproteins.

Inflammatory states induce profound conformational changes in HDL, leading to the replacement of its main apolipoprotein, ApoA-I, with acute-phase proteins such as serum amyloid A. This remodeling disrupts HDL’s normal composition and function, reducing its content of sphingosine-1-phosphate and enriching it with triglyceride-rich lipoproteins (34). As a result, HDL loses its atheroprotective properties – including cholesterol efflux capacity, antioxidant and anti-inflammatory functions – and instead acquires pro-inflammatory characteristics that promote endothelial activation and vascular injury (35, 36).

O’Neill et al. demonstrated the presence of higher levels of anti-HDL and anti-ApoA-I antibodies in SLE patients compared with healthy controls, with titers correlating with disease activity and rising during flares. These antibodies were also associated with lupus nephritis and pathogenic anti-dsDNA levels (37). In psoriasis, Paiva-Lopes et al. reported elevated IgG anti-HDL and anti-ApoA-I antibodies, particularly in patients with more severe disease (38). In early RA, Rodríguez-Carrio et al. found that anti-HDL and anti-ApoA-I antibodies were already elevated, with IgG anti-HDL independently predicting subclinical atherosclerosis (39). Sciascia et al. further showed that antiphospholipid syndrome (APS) patients with arterial thrombosis had significantly higher anti-HDL antibody levels than those with venous events (40). Finally, evidence from a broad range of conditions, including SSc, ANCA-associated vasculitis, and inflammatory bowel disease, has confirmed that anti-HDL antibodies are commonly elevated and strongly associated with impaired HDL antioxidant capacity (41).

Collectively, these findings indicate that humoral autoimmunity against HDL and ApoA-I is a recurrent pattern across autoimmune RMDs, rather than a disease-specific mechanism. Beyond their correlation with disease activity, these antibodies may contribute directly to HDL dysfunction and vascular injury and potentially serve as biomarkers of CV risk in these patient populations. However, data specifically addressing the role of anti-HDL and anti-ApoA-I antibodies in PAD are limited.

### 3.4 Role of pathogenic autoantibodies

#### 3.4.1 Antinuclear antibodies

The antinuclear antibodies (ANA) are among the most important laboratory markers in connective tissue diseases such as SLE, SSc, polymyositis/dermatomyositis, Sjögren’s syndrome (SS), and mixed connective tissue disease (42). In a population-based Polish cohort, ANA positivity was detected in approximately 12% of adults when applying a strict laboratory cutoff, with the highest prevalence observed in women and individuals over 50 years of age (43). Similarly, a nationwide Israeli study found that ANA positivity was independently associated with a 4.6-fold increase in all-cause mortality, even after adjusting for demographic and clinical factors (44).

ANA seropositivity has also been linked to CV atherosclerotic disease and mortality (45). In population studies, ANA have been associated with early markers of vascular stiffness, independently predicting decreased carotid elasticity in young women but not in men (42, 46). Also, cigarette smoking might be one of modifiable external factors that act as trigger for ANA production, and in particular for the anti-DNA antibodies (47).

In PAD, ANA are reported less frequently than in coronary atherosclerosis; approximately 11% of PAD patients show ANA positivity. No significant differences were found between PAD stages in ANA-positive versus ANA-negative groups, although erythrocyte sedimentation rate (ESR) levels were higher in ANA-positive patients (48).

#### 3.4.2 Antiphospholipid antibodies

Antiphospholipid antibodies (aPL) are key immunological markers in connective tissue diseases and represent a key pathogenic link between autoimmunity and vascular disease. Produced by activated B lymphocytes, aPL target phospholipid-binding proteins such as β2-glycoprotein I (β2-GPI, also known as apolipoprotein H), prothrombin, and annexin, as well as cardiolipin, a mitochondrial phospholipid with essential roles in bioenergetics (49). These interactions promote endothelial activation, amplify coagulation pathways, and contribute to a prothrombotic state (50).

Evidence suggests aPL are more prevalent in patients with PAD, particularly in advanced disease stages. Their presence has been associated with greater PAD severity (OR 3.32 for PAD and OR 4.78 for critical limb ischemia) (51), higher overall and CV mortality in IgG anticardiolipin-positive patients (52), and impaired ankle–brachial index (ABI) in those with anti-β2GPI IgG serotypes (53). Similarly, lupus anticoagulant (LA) has been reported up to three to four times more frequently in critical limb ischemia compared with controls (54). In SLE, aPL positivity has also been linked to the early occurrence of PAD, especially in younger patients (55). Together, these findings underscore the central role of aPL in promoting peripheral vascular involvement across autoimmune RMDs.

#### 3.4.3 Rheumatoid factor and anti-cyclic citrullinated peptide antibodies

Rheumatoid factor (RF) and anti-cyclic citrullinated peptide antibodies (anti-CCP), key immunological hallmarks of RA, have been associated with increased CV risk, mainly through their link to higher inflammatory burden and more severe disease phenotypes rather than a clear independent effect. Seropositive patients tend to exhibit greater systemic inflammation and a less favorable cardiometabolic profile, with obesity further amplifying CV risk, particularly in anti-CCP–positive individuals (47, 56). However, evidence regarding an independent association between RF or anti-CCP positivity and CV outcomes remains limited. In a large cohort of postmenopausal women from the Women’s Health Initiative, anti-CCP and RF positivity were not independently associated with CV events after adjustment for traditional risk factors, while markers of inflammation showed stronger associations with outcomes (57). Direct evidence linking RF or anti-CCP positivity specifically to PAD remains limited.

#### 3.4.4 Other emerging autoantibodies

Beyond classical disease-related autoantibodies, novel immune targets have been proposed as biomarkers of atherosclerosis. Antibodies against MYC-associated zinc finger protein (MAZ-Ab) correlate with the number of inflammatory atherosclerotic lesions and with whole-body arterial inflammatory burden, independent of traditional CV risk factors (58, 59). MAZ-Ab may represent part of the autoimmune mechanisms implicated in the pathophysiology of atherosclerosis.

Autoantibodies against G protein–coupled receptors, particularly the β1-adrenergic receptor (β1AR), have also been described. β1AR is expressed on endothelial cells, cardiomyocytes, and fibroblasts. Notably, in individuals younger than 60 years with acute coronary syndrome, reduced β1AR-Ab levels have been associated with a greater likelihood of early re-infarction (60). These findings underscore the potential relevance of antibody-mediated mechanisms beyond classical autoantibody profiles in autoimmune RMDs and PAD.

### 3.5 Role of protective antibodies

#### 3.5.1 Anti-phosphatidylcholine antibodies

Phosphatidylcholine (PC), a major component of LDL particles, becomes exposed following oxidative modification and has been implicated in vascular inflammation. In contrast, anti-PC antibodies, particularly natural IgM, have been shown in multiple studies to exert protective effects against atherosclerosis (61, 62).

Most of the available evidence on anti-PC antibodies comes from studies of coronary and cerebrovascular disease, with no dedicated investigations to date focusing on PAD. In population-based cohorts and in patients with autoimmune diseases, low levels of IgM anti-PC have consistently been linked to an increased CV risk profile. Specifically, reduced anti-PC concentrations correlate with greater carotid intima–media thickness (IMT), a marker of subclinical atherosclerosis (61, 63). These associations suggest that anti-PC antibodies act as protective factors, counteracting the pro-inflammatory and pro-apoptotic effects of oxidized phospholipids.

#### 3.5.2 Antibodies against oxidized LDL

Anti-oxLDL antibodies are among the most studied immunological biomarkers in atherosclerosis. Importantly, their effects appear to depend on antibody isotype. IgM anti-oxLDL antibodies are generally considered protective, being associated with reduced carotid IMT and lower CV risk, whereas IgG antibodies show inconsistent or even adverse associations (64, 65). In RA, elevated IgG anti-oxLDL levels have been linked to greater atherosclerotic burden (66, 67). Data specific to PAD remain limited, although in peritoneal dialysis patients, lower anti-oxLDL levels were independently associated with PAD presence, supporting their potential role as protective biomarkers (68).

#### 3.5.3 Antibodies against apolipoprotein-B

Apolipoprotein B100 (ApoB) is the principal structural component of atherogenic lipoproteins, including LDL, very low-density lipoprotein, intermediate-density lipoprotein, and lipoprotein (a), and reflects the circulating number of atherogenic particles. Unlike LDL-c, ApoB measurement directly reflects the circulating number of atherogenic particles and outperforms LDL-cholesterol as a marker of residual CV risk (69, 70). Evidence is conflicting; in a population-based study, Bertoia et al. observed that higher ApoB levels were associated with increased PAD risk in both sexes, whereas the SURDIAGENE study in type 2 diabetes patients, found no significant relationship between ApoB100 and severe PAD (71, 72).

## 4 PAD: a distinct manifestation of immune-mediated atherosclerosis in RMDs

### 4.1 Burden and underdiagnosis of PAD in RMDs

Most studies in autoimmune rheumatic diseases have primarily focused on coronary or cerebrovascular complications, while data specifically addressing PAD remain limited. Nevertheless, PAD represents a serious vascular manifestation that predicts CV events, increased mortality, and substantial functional impairment. It has been consistently associated with lower-limb pain, reduced mobility, diminished quality of life, and a higher risk of amputation (73). In RA, concomitant PAD has been linked to impaired walking capacity and increased risk of falls (74, 75). Similarly, in SLE, PAD has been associated with higher in-hospital mortality, particularly among younger patients compared with those without PAD (76). Although PAD carries significant prognostic and functional implications, it is likely underdiagnosed in patients with RMDs, with several factors contributing to this underrecognition. Limited mobility and reduced exertion, common in advanced disease or in the presence of musculoskeletal pain and neuropathy, can mask the typical symptom of exertional leg pain and delay diagnosis (77). In addition, vascular and soft tissue calcinosis – a complication affecting up to 20% of patients with SSc – can lead to arterial incompressibility and falsely elevated ABI values, thereby underestimating disease severity (78–80). Evidence on the impact of glucocorticoid therapy on PAD risk and vascular stiffness remains inconclusive. In SLE, long-term glucocorticoid use was associated with lower ABI and increased carotid IMT despite reduced carotid–femoral pulse wave velocity (81). Similarly, in a mixed RMD cohort, glucocorticoid exposure was linked to decreased arterial elasticity without significant changes in ABI (82).

### 4.2 Pathophysiologic particularities of PAD in autoimmune settings

The pathophysiologic mechanisms of PAD in autoimmune rheumatic diseases share core features with atherosclerosis but are amplified by immune dysregulation and chronic inflammation, particularly at the level of endothelial dysfunction, inflammatory activation, and vascular remodeling (21, 28, 83). Among these, several mechanisms have been specifically associated with PAD. Matrix metalloproteinases (MMPs) play an important role in vascular remodeling and have been linked to PAD severity and outcomes. MMP-2 levels are elevated in critical limb ischemia, MMP-10 correlates with mortality, and MMP-2, MMP-3, and MMP-9 increase following endovascular interventions (84, 85). In addition, MMP-7 and MMP-10 have been associated with major adverse CV events in patients with PAD (86). Markers of endothelial activation are also increased in PAD, with elevated plasma E-selectin reflecting enhanced endothelial activity (87). Inflammatory activation is further characterized by increased circulating levels of biomarkers such as hsCRP, fibrinogen, IL-1β, IL-6, tumor necrosis factor-α, intercellular adhesion molecule-1, and vascular cell adhesion molecule (11, 88).

## 5 Disease-specific evidence and clinical implications for PAD in RMDs

### 5.1 Rheumatoid arthritis

RA is the most common chronic systemic inflammatory arthritis, characterized by synovitis, extra-articular manifestations, and the presence of RF and anti-citrullinated protein antibodies (89, 90). PAD in RA has been associated with significant functional impairment, including reduced walking capacity and increased risk of falls (74, 75). Reported prevalence ranges from approximately 4% to over 10%, depending on the population studied and diagnostic method. Both symptomatic and subclinical disease have been described, with several studies demonstrating impaired peripheral arterial function using the ABI (91).

Evidence from case–control studies suggests increased peripheral arterial involvement in RA. In one study, abnormal lower-limb arteries were identified in 19% of patients with RA compared with 5% of controls, with higher rates of arterial incompressibility and obstruction independent of age, sex, and traditional CV risk factors. These differences were most pronounced in patients with advanced joint damage, supporting a potential link between disease severity, inflammation, and PAD (91).

Reduced mobility and musculoskeletal symptoms may mask classical claudication, leading to delayed recognition. Furthermore, PAD is often not systematically assessed in rheumatology practice. Particular attention is warranted in patients with advanced joint destruction, who exhibit increased vascular stiffness and atherosclerotic susceptibility (91, 92).

**Table 1** summarizes 13 clinical studies investigating the association between PAD and RA. Reported prevalence ranges from 4% to over 10%. Large population-based cohorts consistently demonstrated an independent association between the two conditions, whereas smaller case–control studies identified subclinical disease through vascular imaging or ABI assessment. However, most studies primarily examined broader CV outcomes rather than PAD specifically. Methodological heterogeneity, variation in PAD definitions, and under-recognition in clinical practice further limit comparability across studies. Notably, no RA-specific recommendations for PAD screening or prevention currently exist, highlighting an important unmet need.

**Table 1:** Characteristics of clinical studies concerning peripheral artery disease and rheumatoid arthritis.

Author	Study design	Participants	PAD diagnostic method	Key findings
Alkaabi JK., 2003 (93)	Case-control study	40 RA patients, 40 healthy controls	ABI ≤1	ABI <1 in 25% of RA patients vs 2.5% of healthy controls; RA linked to macrovascular diseases; low ABI predicts worse CV mortality.
Bacani AK., 2012 (94)	Inception cohort study	813 RA subjects	ABI ≤0.9 or angiography	37 had noncardiac events (6 PAD, 3 VTE & PAD, 1 cerebrovascular & PAD); incidences similar in both groups.
Bandyopadhyay D., 2019 (95)	Retrospective cohort study	111, 758 RA patients	No direct testing	PAD independently predicts mortality in RA patients with AMI; PAD prevalence ranged from 4.78% to 6.88% in hospitalizations.
Daïen CI., 2019 (96)	Cross-sectional pilot study	200 patients	ABI ≤ 0.9	4.5% of RA patients had undiagnosed PAD; EULAR recommends systematic multimorbidity screening.
del Rincón I., 2004 (97)	Case-control study	647 RA subjects	ABI ≤0.9	13% had lower-limb obstruction; long glucocorticoid use linked to carotid plaque; no relation between lower limb obstruction and glucocorticoids.
Dessein PH., 2015 (98)	Case-control study	151 RA patients, 62 healthy controls	ABI ≤0.9 and arteriography	49.7% had CVD, including 9 PAD; TRAIL density lower in RA; TRAIL linked to heart failure in RA.
Dregan A., 2017 (99)	Cross-sectional & prospective study	502, 641 subjects	No direct testing	RA (28%) linked to higher cardiometabolic events; 52 RA participants had PAD.
González-Meléndez A., 2020 (100)	Cross-sectional study	405 Puerto Rican RA patients	No direct testing	10.6% had arterial events; PAD more common in older males; linked to classical/non-classical factors.
Kurt T., 2015 (101)	Case-control study	90 RA subjects, 80 healthy controls	ABI ≤0.9	Double PAD rates in RA patients; ABI and CIMT assessments recommended for subclinical events.
Sharma TS., 2016 (102)	Retrospective cohort study	1, 266 RA participants	Arterial revascularization	HCQ linked to 72% lower CV risk; 102 CV disease cases, 6 PAD cases required revascularization.
Tehan PE, 2019 (75)	Cross-sectional pilot study	34 RA patients, 38 healthy controls	ABI ≤0.9, TBI ≤0.6, and CWD	RA patients report worse foot pain; controlling RA reduces PAD incidence to match controls.
Wilton KM., 2021 (103)	Longitudinal inception cohort	520 men (260 RA)	No direct testing	RA males with ED had higher PAD risk, but not statistically significant; CV condition worsened by ED.
Wu H., 2025 (104)	Mendelian randomization analysis	14, 361 RA, 43, 923 healthy controls	No direct testing	PAD and RA have a causal association (OR = 1.059, 95% CI: 1.026–1.094, p<0.001)

ABI, Ankle-Brachial Index; ACS, Acute coronary syndrome; AMI, Acute myocardial infarction; CV, Cardiovascular; CIMT, Carotid intima-media thickness; CWD, Continuous wave Doppler; ED, Erectile dysfunction; EULAR, European Alliance of Associations for Rheumatology; HCQ, Hydroxychloroquine; PAD, Peripheral artery disease; RA, Rheumatoid arthritis; TBI, Toe-brachial index; TRAIL, TNF-related apoptosis-inducing ligand; VTE, Venous thromboembolism.

### 5.2 Systemic lupus erythematosus

SLE is a chronic, heterogeneous autoimmune condition characterized by the production of a wide range of autoantibodies and immune complex deposition, which drive multisystem involvement (90). Peripheral arterial involvement is frequently reported, with patients reporting intermittent claudication and lower extremity coldness, occurring more often than in healthy populations and significantly impacting quality of life (105). These manifestations make PAD a serious contributor to disability in SLE (106).

The pathogenesis of PAD in SLE reflects a combination of inflammatory and immune-mediated vascular injury. Deposition of immune complexes can trigger vasculitis with cytotoxic effects, impaired circulation, and tissue ischemia (107). In addition, arterial involvement consistent with PAD may occur early in the disease course, including in younger patients, suggesting a complex arteriopathy potentially involving inflammatory and protease-mediated mechanisms affecting the arterial wall (108).

Reported PAD prevalence in SLE varies widely, but most studies estimate it around 10–13% (107). O’Sullivan et al. reported a ninefold higher incidence of PAD compared with the general population, while Forte et al. described a fourfold greater prevalence than in non-SLE cohorts (106, 109). However, some studies found no significant difference in PAD risk between SLE patients and controls (110).

**Table 2** summarizes 23 studies on PAD in SLE. Reported prevalence is variable, ranging from 1–13% depending on study design and diagnostic method. While some cohorts showed markedly increased PAD incidence, particularly in younger patients, other studies found no significant difference compared with controls. Nevertheless, there is great disparity in PAD definitions and frequent reporting of PAD only as part of composite CV outcomes. Overall, the evidence suggests increased PAD risk in SLE, but dedicated PAD studies are still lacking.

**Table 2:** Characteristics of clinical studies concerning peripheral artery disease and systemic lupus erythematosus.

Author	Study design	Participants	PAD diagnostic method	Key findings
Blachut D., 2024 (111)	Case-control study	98 SLE patients, 68 healthy controls	ABI ≤0.9	Evidence of subclinical atherosclerosis with increased CIMT, arterial stiffness, and atherosclerotic plaques in SLE. ABI and PWV suggest early PAD involvement.
Blazer A., 2017 (112)	Retrospective cohort study	113 African American SLE patients	No direct testing	APOL1 risk alleles associated with atherosclerotic CVD; PAD included in composite outcome.
Chuang YW., 2015 (113)	Case-control study	10, 144 SLE patients, 10, 144 healthy controls	No direct testing	SLE group had 9.39 times higher PAD incidence; highest risk in the first year, more in younger patients (≤34 years).
Erdozain JG., 2014 (114)	Cross-sectional study	216 SLE patients	ABI ≤0.9	74.7% had CV risk factors; 21% with ABI<0.9; no specific SLE features linked to PAD.
Erdozain JG., 2017 (115)	Cross-sectional study	216 SLE subjects	ABI ≤0.9	Increased PAD risk with age.
Erdozain JG., 2020 (116)	Prospective cohort study	216 SLE patients	ABI ≤0.9	18 AVEs (2 PAD) found; abnormal ABI noted in 24.1%, particularly men; carotid atherosclerosis linked to higher risks.
Fernández M., 2007 (117)	Multiethnic longitudinal study	72 postmenopausal SLE subjects	No direct testing	Non-HRT users had more vascular cases; estrogens not significant.
Fernández-Nebro A., 2015 (118)	Cross-sectional study	3658 Spanish SLE subjects	No direct testing	10.9% experienced stroke, AMI, angina, or PAD; PAD prevalence noted at 2.2%.
Hassan AA., 2013 (119)	Case control study	100 SLE patients, 100 healthy controls	ABI ≤0.9	Increased PAD frequency in SLE linked to high steroid doses and active disease.
Ho KT., 2005 (120)	Multiethnic longitudinal study	442 SLE subjects with aPL antibodies	No direct testing	Disease activity and smoking significant for arterial and venous events; aPL antibodies not linked to thrombosis onset.
Hsu CY., 2017 (121)	Prospective cohort study	8397 SLE subjects	No direct testing	Similar vascular occurrence in HCQ users vs controls; longterm HCQ treatment not protective for PAD in SLE.
Katz G., 2019 (122)	Case-control study	252, 676 SLE patients, 758, 034 healthy controls	No direct testing	SLE linked to higher atherosclerotic disease (25.6% vs. 19.2%); PAD risk higher (4.6% vs. 3.7%), peaking at ages 30-49.
Koenig KF., 2015 (123)	Cross-sectional study	241 SLE subjects, 193 T1DM subjects	Doppler US or angiography	PAD incidence: SLE 1.2%, T1DM 5.6%; comparable vascular manifestations in both groups.
Lundström E., 2013 (124)	Case control study	665 SLE Caucasian subjects, 1403 healthy controls	Doppler US or angiography	HLA-DRB1 genes correlated with vascular phenomena; ischemic PVD prevalence varies by genotype.
McDonald J., 1992 (125)	Case control study	563 SLE patients	Doppler US or angiography	10 cases of PAD, younger ages; no significant differences in traditional risk factors.
Petri MA., 2019 (126)	Prospective cohort study	1721 SLE subjects	No direct testing	SLE-related risk factors led to increased 10-year CV risk; 168 CV events reported.
Toloza SMA., 2004 (127)	Longitudinal cohort study	546 SLE subjects across 3 ethnic groups	No direct testing	Older age, smoking, high hs-CRP, and aPL predicted vascular events in SLE; outcomes included PAD.
Tselios K., 2020 (128)	Longitudinal cohort study	1532 SLE patients based on BP	Arterial revascularization	124 AVEs over 2 years; SLE and hypertension significantly linked to AVEs.
Tziomalos K., 2017 (129)	Case control study	55 SLE patients, 61 controls	ABI ≤0.9	Similar PAD frequency; ABI not associated with SLE clinical/biological criteria.
Urowitz MB., 2010 (130)	Longitudinal inception cohort study	1, 249 SLE patients	Angiography	72 CVD events; PAD was rarest. Risk factors included age at SLE diagnosis, male gender, obesity, and smoking.
Wigren M., 2018 (131)	Case control study	484 SLE subjects, 253 healthy controls	No direct testing	Biomarkers of apoptosis, inflammation, and tissue degradation elevated in SLE; associated with CVD including PAD.
Ye Y., 2015 (132)	Case control study	135 SLE subjects, 135 healthy controls	ABI ≤0.9	Increased interarm BP difference in SLE, predictive of PAD; High variation linked to longterm steroids.

ABI, Ankle-brachial index; AMI, Acute myocardial infarction; APOL1, Apolipoprotein L1; AVE, Acute vascular event; BP, Blood pressure; CIMT, Carotid intima-media thickness; CV, Cardiovascular; CVD, Cardiovascular disease; EPCs, Endothelial progenitor cells; HCQ, Hydroxychloroquine; HLA-DRB1, Human leukocyte antigen DR beta 1; hs-CRP, High-sensitivity C-reactive protein; IL-6, Interleukin 6; IMT, Intima-media thickness; MTHFR, Methylenetetrahydrofolate reductase; PAD, Peripheral artery disease, PVD; Peripheral vascular disease; RA, Rheumatoid arthritis; SLE, Systemic lupus erythematosus; T1DM, Type 1 diabetes mellitus; US, Ultrasound; TGF-β1, Transforming growth factor beta 1.

### 5.3 Antiphospholipid antibody syndrome

APS is an autoimmune disease defined by the presence of aPL, including anti-β2GPI, aCL, and LA, which bind to plasma proteins with an affinity for phospholipid surfaces. The incidence of APS is estimated at 2.1 per 100, 000 people, with a prevalence of about 50 per 100, 000 people and similar frequencies across sex groups (49). APS may occur as a primary condition or secondary to other autoimmune diseases, most commonly SLE, with approximately 40% of patients with SLE having aPL. Over time, a significant proportion of patients with SLE and persistent aPL may progress to APS (133). Clinically, SLE-associated APS has been linked to a more severe disease phenotype, with increased frequency of major organ involvement (134).

The increased CV risk related to APS is closely associated with traditional CVD risk factors, as well as factors such as oral estrogen use and smoking in this population (49, 135). Peripheral arterial involvement in APS may arise through two distinct but overlapping mechanisms, including both thrombotic occlusion and accelerated atherosclerosis. Thrombotic events represent the first mechanism, with aPL causing endothelial activation, platelet aggregation, and coagulation pathway activation. In this context, limb ischemia may occur independently of underlying atherosclerotic plaque formation (136). In parallel, aPL have also been implicated in vascular inflammation and endothelial dysfunction, potentially contributing to atherosclerotic processes. These effects may be mediated directly through the proinflammatory and procoagulant activity of aPL on endothelial cells, or indirectly via immune mechanisms involved in autoantibody-mediated thrombosis (25).

Non-criteria antiphospholipid antibodies, such as antiphosphatidylserine and antiphosphatidylinositol antibodies, have been associated with increased intima–media thickness and carotid artery stenosis, suggesting a broader role of humoral autoimmunity in vascular involvement, although their direct relationship with PAD remains insufficiently defined (137).

**Table 3** summarizes 5 studies on PAD in APS. The evolution of PAD in this patient group can be severe, leading to amputations, with lower limb vessels most often involved. Abnormal ABI and higher aGAPSS scores have been linked to PAD. Overall, PAD is highly relevant in APS but still remains under-studied and often reported only within composite CV outcomes.

**Table 3:** Characteristics of clinical studies concerning peripheral artery disease and antiphospholipid syndrome.

Author	Study design	Participants	PAD diagnostic method	Key findings
Asherson RA., 2007 (138)	Retrospective case series	21 patients with high aPL	Amputation	Severe PAD leading to amputations.
Asherson RA., 2008 (139)	Case-report	21 patients with SLE, APS, lupus-like pathology	Amputation	PAD in APS may lead to limb amputation; primary cause difficult to determine without histology.
Bucci T., 2022 (140)	Cross-sectional study	100 APS patients	ABI ≤0.9	60 had VTE, 40 had ATE; low ABI (19%) suggests ATE link.
Di Minno MND., 2018 (141)	Prospective cohort study	192 aPL patients	No direct testing	Rising aGAPSS scores linked with increased PAD.
Hinojosa CA., 2017 (142)	Retrospective cohort study	807 APS patients	Amputation	APS involved mainly lower limb vessels; arterial regeneration feasible despite high morbidity.

ABI, Ankle-brachial index; aGAPSS, Adjusted Global Antiphospholipid Syndrome Score; aPL, Antiphospholipid antibodies; APS, Antiphospholipid syndrome; ATE, Arterial thromboembolism; PAD, Peripheral artery disease; VTE, Venous thromboembolism.

### 5.4 Systemic sclerosis

SSc is a severe autoimmune disorder characterized by vasculopathy and organ fibrosis. CVD is associated with poor prognosis and accounts for approximately 26% of scleroderma-related mortality. Coronary atherosclerosis, PAD, and cerebrovascular calcification are increased in SSc. However, compared with RA and SLE, atherosclerosis in SSc appears to be less strongly driven by systemic inflammation (90).

Peripheral arterial involvement in SSc is increasingly recognized, with evidence of both macrovascular and distal vascular disease. Early observations linked SSc to peripheral macrovascular complications, including lower limb amputation. Macrovascular involvement has been reported in up to 58% of patients, with 16% requiring lower extremity amputation and approximately 21.7% experiencing intermittent claudication (143). At the digital level, distal PAD is more common than expected. Although not consistently reported in SSc, generalized early atherosclerotic lesions are present (144).

The pathophysiology of PAD in SSc reflects a combination of endothelial dysfunction, vascular remodeling, and immune-mediated injury. Both microvascular and macrovascular involvement contribute to impaired perfusion and tissue hypoxia. Mechanisms include endothelial injury, dysregulation of coagulation and fibrinolysis, and the presence of anti-endothelial cell antibodies, all of which promote vascular occlusion and structural vascular damage (25, 145, 146).

**Table 4** summarizes 18 studies on PAD in SSc, with most studies confirming increased macrovascular involvement, including higher PAD incidence, arterial occlusion, and risk of digital and limb amputations. However, there is heterogeneity in definitions, small sample sizes, and PAD is commonly reported only in composite vascular outcomes. Overall, evidence indicates SSc carries an elevated PAD risk, but its true prevalence and clinical course remain underexplored.

**Table 4:** Characteristics of the clinical studies concerning peripheral artery disease and systemic sclerosis.

Author	Study design	Participants	PAD diagnostic method	Key findings
Arhuidese I., 2016 (147)	Retrospective cohort study	18 SSc, 410 non-SSc with PAD	ABI ≤0.9, duplex US, digital pressure measurement	SSc patients with PAD had seven times higher graft failure rates post-bypass surgery.
Badak SO., 2021 (148)	Cross-sectional study	88 SSc patients	Doppler US	55.7% had increased CIMT linked to disease duration; plaques in 67.7%; arterial occlusion detected in peripheral vessels.
Caramaschi P., 2012 (149)	Retrospective cohort study	188 SSc patients (162 women)	Doppler US	4.8% of women underwent surgical digital amputations. Digital amputations linked to PAD, hypercholesterolemia, RP, and ACA.
Caramaschi P., 2012 (150)	Retrospective cohort study	115 SSc subjects on iloprost	Doppler US	2 developed gangrene requiring amputation; iloprost may slow vasculopathy.
Cassius C., 2021 (151)	Prospective cohort study	86 SSc patients	ABI ≤ 0.9, TBI < 0.75, duplex US	76% had lower limb perfusion issues, suggesting PAD underdiagnosed with ABI alone.
Emad Y., 2014 (152)	Case-control/cross-sectional pilot study	22 SSc patients	Angiography	SSc linked to upper limb macrovascular vasculopathy; Raynaud’s present in all, no PAD risk factors.
Ho M., 2000 (153)	Case-control study	54 SSc, 43 healthy controls	ABI <0.9	17% had PAD; more prevalent macrovascular disease.
Hsieh MC., 2021 (154)	Case-control study	1106 SSc patients, 4424 healthy controls	No direct testing	Higher PAD incidence in SSc (7% vs. 3%), even without comorbidities.
Nordin A., 2013 (155)	Case-control study	111 SSc, 105 healthy controls	ABI ≤0.9	Higher ischemic events in SSc with ACA, despite similar ABI and IMT.
Stafford L., 1998 (156)	Retrospective cohort study	20 SSc, 20 healthy controls	Doppler US	No significant lower limb arterial differences; 3 patients had limb loss.
Veale DJ., 1995 (157)	Prospective pilot cohort study	53 SSc patients	No direct testing	21.7% reported calf pain; linked to macroarteriopathy and angina.
Wig S., 2014 (158)	Longitudinal retrospective cohort study	200 SSc patients (83% women)	ABI ≤0.9	ABI stable over 8 years; decreased ABI linked with age, limited SSc, and ACA positivity.
Yen T-H., 2024 (159)	Nationwide cohort study	1379 SSc patients, 2758 healthy controls	No direct testing	Increased risk of PAD in SSc patients (IRR 3.67; 95% CI 2.84–4.74).
Zeng Y., 2012 (160)	Cross-sectional study	48 SSc, 46 healthy controls	ABI ≤0.9	Lower ABI in SSc, higher sIAD; linked to PAD risk.

ABI, Ankle-brachial index; ACA, Anticardiolipin antibodies; Aix, Augmentation index; CIMT, carotid intima-media thickness; CI, Confidence interval; CVD, Cardiovascular disease; IMT, Intima-media thickness; IRR, Incidence rate ratio; PAD, Peripheral artery disease; RP, Raynaud’s phenomenon; SSc, Systemic sclerosis; US, Ultrasound; sIAD, Systolic interarm difference.

### 5.5 Polymyalgia rheumatica

Polymyalgia rheumatica (PMR) is the most prevalent inflammatory rheumatic disorder in adults over 50. It is characterized by morning stiffness and aching in the neck, shoulders, and hips lasting at least 30 minutes for a minimum of one month, accompanied by systemic symptoms due to IL-6 release, laboratory evidence of inflammation (elevated ESR and CRP), and a rapid response to low-dose glucocorticoids, which supports the diagnosis (161, 162).

Patients with PMR have an increased burden of cardiovascular comorbidities, including myocardial infarction (MI), PAD, and cerebrovascular disorders, which contribute substantially to healthcare costs (163). The elevated risk of PAD may be linked to the persistent systemic inflammatory burden associated with PMR, although its etiology and overlap with giant cell arteritis remain largely unclear (164, 165). Chronic inflammation and endothelial dysfunction in PMR may exacerbate claudication and ischemia, predisposing patients to vascular events (166). The heightened risk of atherosclerotic PAD in PMR can be explained by arterial blockages reflecting (subclinical) vasculitis and sustained inflammation promoting early atherosclerosis (167). PMR has therefore been described as a proatherogenic condition, with carotid IMT serving as a marker of premature atherosclerosis and a prognostic tool, given that each 1% increase in flow-mediated dilation has been associated with a 13% reduction in the probability of future CV events. Moreover, the fact that patients with PMR often require approximately six months of steroid therapy before achieving significant recovery highlights how prolonged inflammatory activity may contribute to increased CV risk (165).

**Table 5** details clinical studies highlighting the main findings in patients with PAD and PMR. Overall, PAD prevalence appears higher in PMR compared with controls. Subclinical vascular changes such as increased carotid IMT, arterial stiffness, and aortic dilatation have been reported, even when ABI remains normal. Evidence is limited, heterogeneous, and often confounded by steroid exposure, but it suggests that PMR is a proatherogenic condition with increasing PAD risk.

**Table 5:** Characteristics of clinical studies concerning peripheral artery disease and polymyalgia rheumatica.

Author	Study design	Participants	PAD diagnostic method	Key findings
Mazzantini M., 2012 (168)	Retrospective observational review	222 PMR patients	Clinical diagnosis, amputation	43% experienced adverse events (PAD, CVA, AMI) after 31 months of low-dose corticosteroids.
Pujades-Rodriguez M., 2020 (169)	Longitudinal cohort study	87, 794 adults with autoimmune diseases	No direct testing	Glucocorticoid use increased PAD risk in a dose-dependent manner.
Scrivo R., 2020 (170)	Cross-sectional study	48 PMR patients with CV risk factors, 56 controls with CV risk factors	ABI ≤0.9	PMR patients showed increased carotid IMT, arterial stiffness, and aortic diameter, but no difference in ABI.
Warrington KJ., 2009 (164)	Retrospective cohort study	353 PMR patients, 705 healthy controls	ABI ≤0.9	8.5% of PMR patients had PAD vs. 4.1% controls; linked to chronic inflammation, not GCA.

AMI, Acute myocardial infarction; CAVI, Cardio-ankle vascular index; CVA, Cerebrovascular accident; CV, Cardiovascular, CVD; Cardiovascular disease; GCA, Giant cell arteritis; IMT, Intima-media thickness; PAD, Peripheral artery disease; PMR, Polymyalgia rheumatica.

### 5.6 Psoriatic arthritis

Psoriatic arthritis (PsA) is a chronic inflammatory arthritis classified as a form of spondyloarthritis, typically occurring in association with psoriasis. It presents with a broad spectrum of manifestations, the most common being synovitis (95%), followed by dactylitis, enthesitis, and less frequently, axial involvement (5%) (171).

Adults with PsA have a 55% higher risk than the general population of CV events, including MI, cerebrovascular disease, and heart failure, which are significantly more common. In addition, individuals with PsA appear to be at greater risk of CVD than those with psoriasis alone. It has been proposed that the increased extra-articular manifestations are partly driven by the chronic inflammatory state characteristic of PsA (171). However, the role of traditional CV risk factors to account for CVD burden is higher in PsA compared to other forms of inflammatory arthritis, such as RA (172, 173). This notion creates a specific scenario for CV risk management in this condition (174).

Insulin resistance results from systemic inflammation associated with obesity and metabolic syndrome, shifting the balance of insulin’s pro- and anti-atherogenic actions toward the former. This promotes endothelial dysfunction and creates the conditions for atherosclerosis and major CV events such as MI or stroke (161, 175). When age and sex were considered, one study revealed that patients with PsA had a 60% higher prevalence of PAD compared with controls (175).

**Table 6** summarizes the clinical studies reporting PAD in patients with PsA. Evidence shows PAD prevalence and incidence are higher in PsA compared with controls, ranging from 2.8% to nearly 5% in large cohorts. Subclinical disease is also common, with up to 23.5% of patients showing borderline ABI values, particularly in those with longer disease duration and higher activity. These findings support that PsA contributes to PAD risk. However, data are still limited, and PAD in PsA is often under-recognized, while the need for more PsA-PAD oriented studies is imperative.

**Table 6:** Characteristics of clinical studies concerning peripheral artery disease and psoriatic arthritis.

Author	Study design	Participants	PAD diagnostic method	Key findings
Bilim S., 2021 (176)	Case-control study	51 PsA patients (27 women), 50 healthy controls	ABI ≤0.9	Lower ABI in PsA group; 23.5% had borderline ABI, linked to longer disease duration and activity; ABI useful for detecting subclinical atherosclerosis.
Kaine J., 2019 (177)	Retrospective cohort study	14, 898 PsA patients and 35037 healthy controls	No direct testing	Higher PAD incidence in PsA (2.8%) vs. controls (2.2%); PsA linked to CVD risk, more comorbidities, and hospitalizations.
Kibari A., 2019 (178)	Retrospective cross-sectional case-control	3, 161 PsA patients (53.4% female), 31, 610 healthy controls	No direct testing	PsA strongly associated with CVD and PAD; PAD prevalence higher in PsA (4.9%) than controls (3.4%).

ABI, Ankle-brachial index; AS, Ankylosing spondylitis; CVD, Cardiovascular disease; PAD, Peripheral artery disease; PsA, Psoriatic arthritis.

### 5.7 Sjögren’s syndrome

Sjögren’s syndrome (SS) is a common autoimmune disorder characterized by systemic inflammation and B-cell–mediated immune activation (90). PAD in SS remains underdiagnosed and insufficiently characterized. Most studies have focused on surrogate markers of vascular involvement, including arterial stiffness and subclinical atherosclerosis, rather than clinically defined PAD. Vascular involvement has been increasingly recognized in SS (21).

Autoimmune mechanisms may contribute to vascular dysfunction. In particular, autoantibodies against lipoprotein lipase have been described and are associated with elevated triglycerides and features of accelerated atherosclerosis (25). Chronic inflammation and cytokine-mediated pathways may further promote vascular alterations; however, their direct role in PAD development remains uncertain (21).

Evidence linking SS to PAD remains limited and mostly indirect (**Table 7**). PAD remains underdiagnosed and understudied in pSS.

**Table 7:** Characteristics of the clinical studies concerning peripheral artery disease and Sjögren’s syndrome.

Author	Study design	Participants	PAD diagnostic method	Key findings
Bohman R., 2024 (179)	Cohort study	111 SS, 194 healthy controls	No direct testing	High ESSDAI (≥5) linked to increased odds of PVD.
Santos SC, 2023 (180)	Retrospective cohort study	102 SS patients (82% female)	No direct testing	7% had PAD
Yu K., 2021 (181)	Longitudinal cohort study	535 adults with moderate/severe DED (81.1% female)	No direct testing	Severe DED linked to systemic diseases such as SS and PAD, with smoking and dyslipidemia increasing severity.

DED, Dry eye disease; ESSDAI, EULAR Sjögren’s syndrome disease activity index; PAD, Peripheral artery disease; PVD, Peripheral vascular disease; SS, Sjögren’s syndrome.

## 6 Therapeutic and preventive perspectives

### 6.1 Lipid-lowering and anti-atherosclerotic therapy

Patients with RMDs and concomitant PAD should be considered at very high CV risk and managed accordingly. Statin therapy remains the cornerstone of treatment, both for its lipid-lowering properties and for its immunomodulatory effects, achieved through inhibition of the mevalonate pathway (182). Across autoimmune diseases, several studies have highlighted pleiotropic benefits. In SLE, statins reduce cytokine production, with some evidence of lowering CRP levels depending on the agent used, although without consistent improvement in disease activity scores (183). In RA, a meta-analysis of 19 studies reported significant decreases in CRP, ESR, and the 28-joint disease activity score (184). In SSc, statin therapy has been associated with reduced IL-6 levels and clinical improvement in Raynaud’s phenomenon and digital ulcers, without cases of statin intolerance – an often overstated concern in RMD populations (185).

By contrast, a meta-analysis of 10 randomized trials in PsA found no statistically significant improvement in disease parameters with statin use (186). Finally, in APS, statins may exert additional antithrombotic effects through anti-aggregant mechanisms, supported by preclinical and small clinical studies, but warranting further investigation (187).

These results highlight the neutral, if not beneficial impact of statins in this population. Statin intolerance is often overdiagnosed in this patient group, owing to the baseline increased serum levels of creatine kinase. However, physicians should be vigilant, as this does not represent true statin intolerance and discontinuation is rarely necessary (188–190). Most patients tolerate rechallenge, continuation at a lower dose, a change from one type of statin to another or combination therapy of lower dose of statin with ezetimibe/non-statin drug (191, 192). In fact, in a population-based cohort, statin therapy reduced mortality by 16% among patients with SLE, SS, and SSc (193).

Ezetimibe is a useful therapeutic option in RMDs, either as an add-on to statins or as monotherapy in cases of statin intolerance (188). In SLE, combined therapy with pravastatin and ezetimibe led to reductions in carotid IMT and CRP levels, although the study by Vestra-Lastra et al. included a small cohort and warrants confirmation in larger populations (194). Similarly, in a multicenter open-label trial in RA, rosuvastatin plus ezetimibe was shown to be safe, producing significant LDL-c reductions but no clear effects on inflammatory markers (195).

There are no direct data on the use of bempedoic acid (BA) in RMDs patients; however, considering that BA is a prodrug in muscles activated in hepatocytes, that it acts via the AMP-activated protein kinase pathway, that it is associated with a significant (>20%) reduction in hsCRP, and that the CLEAR Outcomes study showed high efficacy (a 13% reduction in the primary composite CVD outcome), its use in patients with RMDs who are at high or very high CV risk should always be considered (192).

Evidence in other RMDs remains limited. For PsA, no clinical data are available to date, although experimental work suggests potential benefit (196). For the remaining RMDs discussed, bibliographic data are scarce, underscoring the need for further studies.

Evidence on the effect of fibrates on RMDs is very scarce. Studies suggest that fibrates decrease ESR and CRP levels in RA patients, as well as disease activity (197, 198). No data is available for the remaining RMDs.

Proprotein convertase subtilisin/kexin type 9 (PCSK9) regulates LDL receptor activity and thereby controls circulating ApoB-containing lipoproteins. PCSK9 inhibitors lower LDL-c and improve CV outcomes, including in patients with severe PAD (199, 200).

In the FOURIER trial, PCSK9 inhibition markedly reduced LDL-C levels and led to a 58% reduction in major CV events, comprising MI, stroke, and death due to CV causes, in patients with inflammatory arthritis, including RA and PsA (201). Beyond lipid lowering, PCSK9 may also influence immune and inflammatory pathways. In a study by Frostegård et al., RA patients with lower baseline PCSK9 concentrations demonstrated better clinical outcomes and greater responsiveness to TNF-α inhibitors, suggesting that PCSK9 inhibition might modulate inflammatory disease activity itself (63).

### 6.2 Emerging anti-inflammatory therapeutic approaches

Colchicine and small molecules are being evaluated for inflammasome inhibition (202, 203). Colchicine interferes with the assembly of microtubules and thus dysregulates multiple inflammasome components. When added to standard secondary prevention therapy, colchicine has demonstrated protective effects in patients with stable CAD (202). The NLRP3 inflammasome is a macromolecular structure and a primary driver of sterile inflammation in response to myocardial ischemia (203). The NLRP3 inflammasome is composed of the NLRP3 protein, apoptosis-associated speck-like proteins containing a caspase recruitment domain, and pro–caspase-1 (204).

Inhibitors of the NLRP3 inflammasome are also being evaluated in the context of atherosclerotic CVD. NLRP3 inflammasome inhibitors include OLT1177 (Dapansutrile) (69), MCC950 (205), and CY-09 (204). In this regard, OLT1177 (dapansutrile) has been shown to reduce myocardial infarct size while also preserving contractile function after reperfusion (203, 206). MCC950 has demonstrated good efficacy in reducing inflammation in both autoimmune diseases and CV and metabolic diseases (205). Furthermore, CY-09 may effectively reduce fatty acid synthesis and lipid peroxidation (204).

Recent therapeutic developments in immune rheumatic diseases include the use of antibodies targeting cytokines. In particular, for RA, Still’s disease, spondyloarthropathies, and PsA, many valuable drugs targeting key cytokines of the systemic inflammatory response have been introduced into daily practice (26). However, PAD-specific data are lacking.

## 7 Conclusion

Autoimmune systemic diseases are accompanied by an increased CV risk, distinct from that conferred by traditional risk factors. This excess risk may be explained by a complex interplay between persistent inflammation and immune dysregulation, which amplify the effects of conventional risk factors, especially lipoprotein metabolism.

PAD is most consistently reported in RA and SLE, with supportive but less extensive evidence in APS and SSc, and only limited data in PsA, pSS, and PMR. Autoantibodies and immune-mediated pathways appear to contribute to vascular involvement; however, their role in PAD specifically remains insufficiently defined. While certain antibody profiles have been associated with surrogate markers of vascular disease, current evidence does not support their use in routine PAD screening. Nevertheless, they may represent potential tools for risk stratification in selected populations, a hypothesis that requires validation in prospective studies. In this context, future studies should clarify whether autoantibodies directly contribute to vascular injury or simply reflect underlying systemic inflammation, and whether they can improve risk stratification beyond traditional CV risk factors.

From a therapeutic perspective, patients with RMDs and PAD are generally considered at high or very high CV risk. Lipid-lowering therapies, particularly statins, are widely used and appear to be safe in this population, although evidence specifically addressing PAD outcomes in RMDs remains limited. Additional agents such as ezetimibe and PCSK9 inhibitors may be considered when lipid targets are not achieved, while the potential vascular impact of biologic and anti-inflammatory therapies requires further investigation. A combined approach addressing both CV risk factors and inflammation may be relevant, although its clinical impact remains to be established.

Several key gaps remain. First, PAD is frequently underdiagnosed in RMDs, partly due to atypical presentation and limited systematic screening. Second, most available data derive from studies focused on coronary or cerebrovascular outcomes, with PAD often reported only as part of composite endpoints. Future research should prioritize PAD-specific endpoints, standardized diagnostic approaches, and longitudinal studies to clarify risk stratification and guide management in this population. Additionally, whether selected high-risk RMD populations could benefit from more systematic vascular assessment remains an open question that warrants prospective evaluation.
